# Supplementary material for: Whole-Genome Sequencing Can Identify Clinically Relevant Variants from a Single Sub-Punch of a Dried Blood Spot Specimen
Source: Int J Neonatal Screen. 2023 Sep 21;9(3):52. doi: 10.3390/ijns9030052 (PMC10532340; doi:10.3390/ijns9030052)
Supplement: Supplementary file 1 [file IJNS-09-00052-s001.zip › IJNS-2595939-supplementary.pdf]

Supplementary Materials

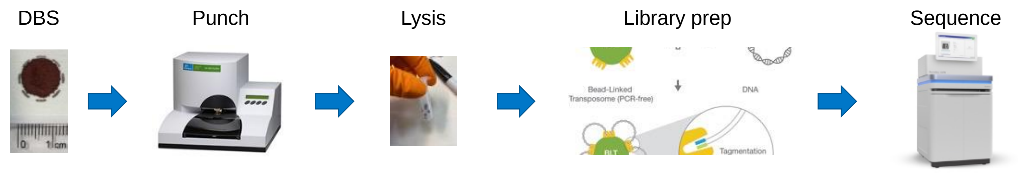

Figure S1. Overview of the DBS input workflow. A sub-punch of the DBSs was taken using a DBS puncher and incubated with lysis buffer. DNA was recovered from the lysate by bead purification, then used as an input for transposon-based library prep. The resultant libraries were sequenced on a NovaSeq 6000.

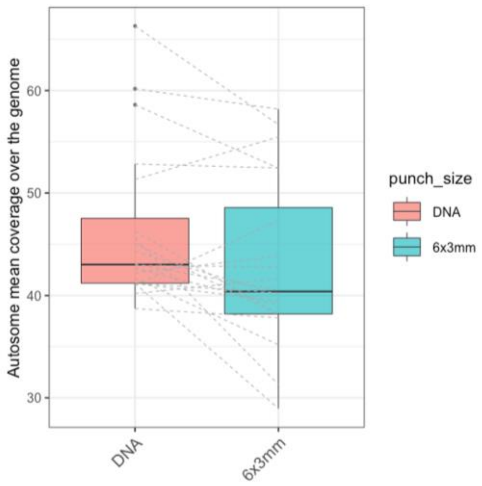

Figure S2. Performance of extracted DNA vs. DBSs across the 40 participants. Average autosomal coverage over the genome achieved across the 40 samples (median = 44.6×, range = 29.0×–66.3×). Data obtained from the same participant are linked by dotted lines.

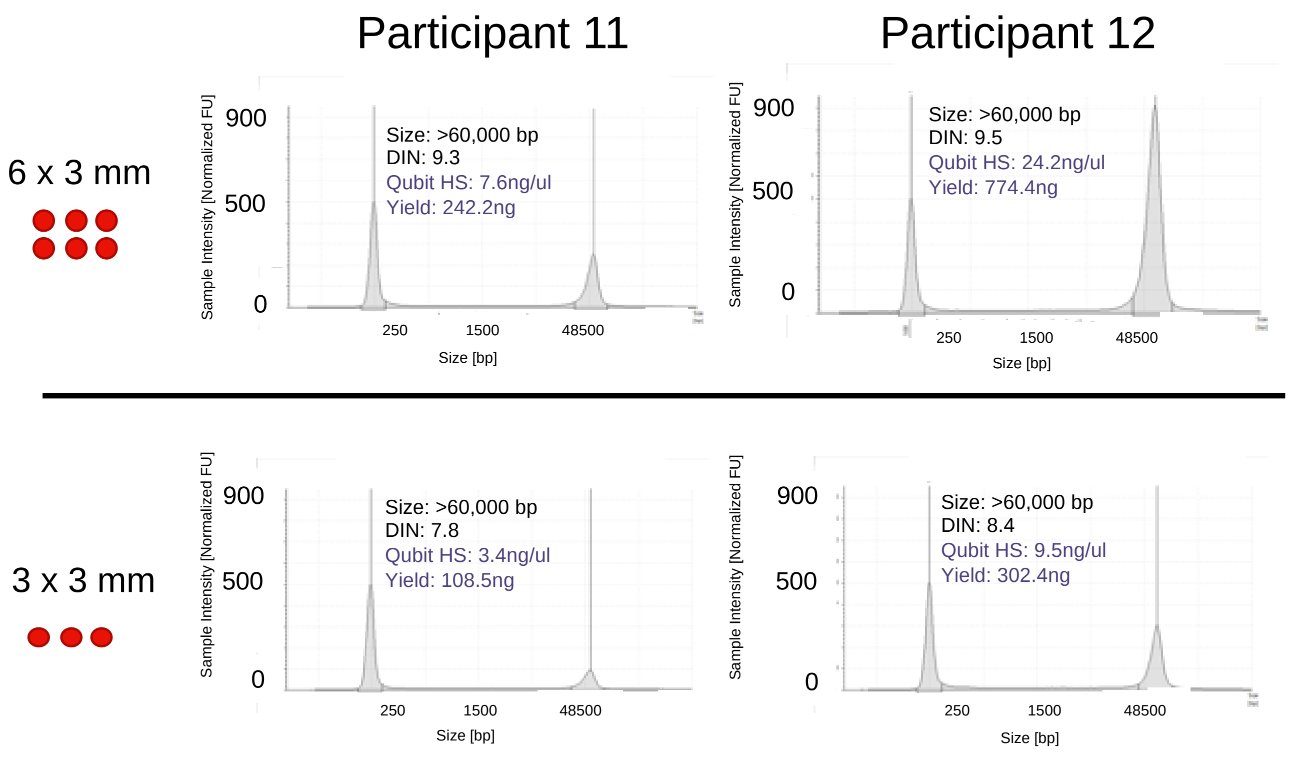

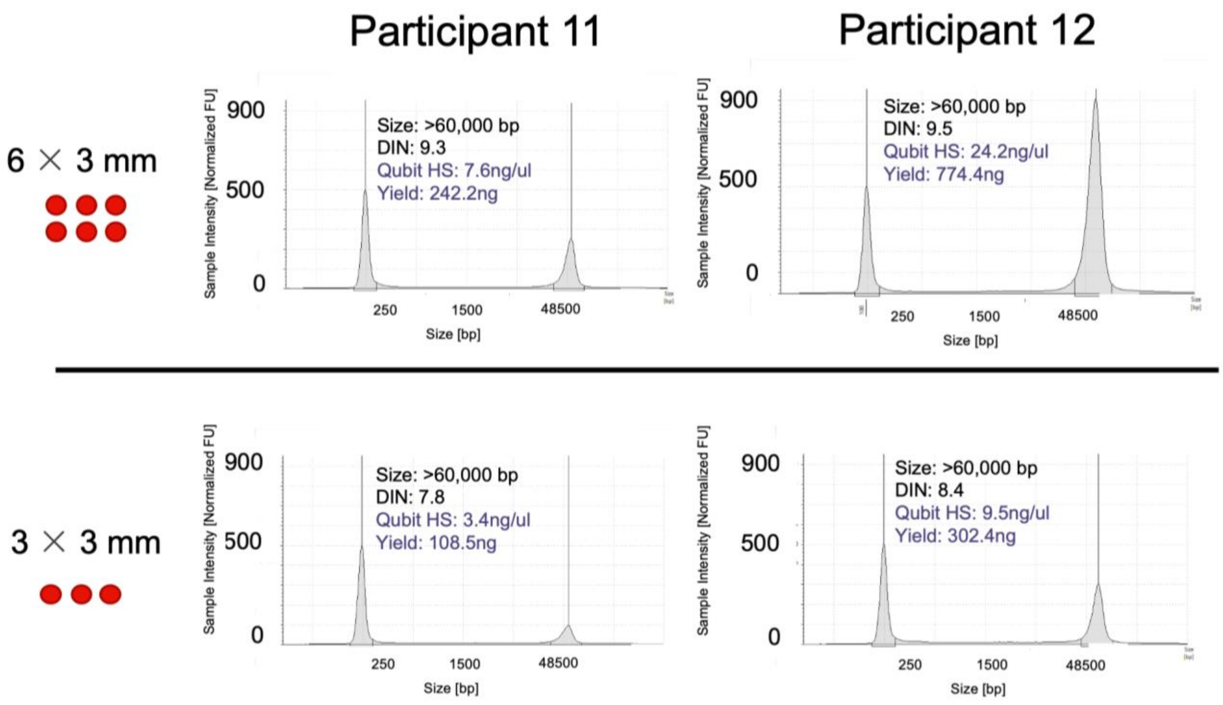

Figure S3. High-quality DNA is available as an input for library preparation from the Illumina Lysis Reagent kit. Remaining lysate material from 6 × 3 mm and 3 × 3 mm library preparation reactions for 2 samples were run on Agilent TapeStation. Y axis: sample intensity (normalized fluorescence units), X axis: predicted size of molecules in base pairs. DIN: DNA integrity number output from Agilent software. Leftmost peak in all traces is the supplied control marker; right peak is genomic DNA. All samples result in fragments of >60 Kbp. DNA concentration, as determined by Qubit HS assay, and resultant calculated input for the library preparation assay shown for comparison.
